# Effect of glycerol on properties of chitosan/chlorhexidine membranes and antibacterial activity against *Streptococcus mutans*

**DOI:** 10.3389/fmicb.2024.1430954

**Published:** 2024-08-15

**Authors:** José Alberto Hachity-Ortega, Alberto V. Jerezano-Domínguez, Laura Abisai Pazos-Rojas, Abigailt Flores-Ledesma, Diana del C. Pazos-Guarneros, Karla Aimée Parra-Solar, Eric Reyes-Cervantes, Ismael Juárez-Díaz, Manuel E. Medina, Mayra González-Martínez, Brenda Eréndida Castillo-Silva, Beatriz Xochitl Ávila-Curiel, Jesús Hernández-Juárez, América Rivera-Urbalejo, Paola G. Gordillo-Guerra, Miguel Angel Casillas-Santana

**Affiliations:** ^1^Facultad de Odontología, Universidad Autónoma “Benito Juárez” de Oaxaca, Oaxaca, Mexico; ^2^Facultad de Estomatología, Benemérita Universidad Autónoma de Puebla, Puebla, Mexico; ^3^Tecnológico de Monterrey, Escuela de Ingeniería y Ciencias, Puebla, Mexico; ^4^Facultad de Ingeniería y Ciencias Químicas, Benemérita Universidad Autónoma de Puebla, Puebla, Mexico; ^5^Direccción de Innovación y Transferencia de Conocimiento, Benemérita Universidad Autónoma de Puebla, Puebla, Mexico; ^6^Centro de Investigación en Micología Aplicada, Universidad Veracruzana, Veracruz, Mexico; ^7^CONAHCyT-Centro Interdisciplinario de Investigación para el Desarrollo Integral Regional, Unidad Oaxaca, Oaxaca, Mexico; ^8^Survival of Microorganism, Laboratorio de Ecología Molecular Microbiana (LEMM), Centro de Investigaciones en Ciencias Microbiológicas (CICM), Instituto de Ciencias (IC), Benemérita Universidad Autónoma de Puebla (BUAP), Puebla, Mexico; ^9^Departamento de Sistemas Biológicos, Unidad Xochimilco, Universidad Autónoma Metropolitana, Ciudad de Mexico, Mexico

**Keywords:** chitosan, glycerol, chlorhexidine, membrane, antibacterial, *S. mutans*

## Abstract

**Introduction:**

Chitosan membranes with glycerol can function as an effective dispersing agent for different antibiotics or active ingredients that can be used in the treatment of diseases present in the oral cavity.

**Methods:**

The effects of the addition of glycerol on the mechanical, water absorption, swelling, pH, thickness, disintegration, rugosity, and antibacterial properties of chitosan-chlorhexidine- glycerol membranes were investigated in this study.

**Results and discussion:**

Mechanical results indicated that chitosan membranes' rugosity, strength, flexion, and thickness differed at loading 1, 3, 5, 10, 15, and 20% of glycerol (*p* < 0.05). The chitosan membranes' rugosity, dissolution, strength, and pH results were significantly enhanced by the presence of glycerol at 3, 5, and 10% concentrations. In this investigation, the antimicrobial activity model used was the inhibition of *Streptococcus mutans* CDBB-B-1455 by chitosan-chlorhexidine membranes. It was observed that there was no change in inhibition with different concentrations of glycerol. The results suggest that chitosan-glycerol-chlorhexidine membranes may be a potential candidate for topical antiseptic application in buccal-dental disorders caused by *S. mutans*, such as caries, periodontal diseases, and oral squamous cell carcinoma, helping to prevent the development of serious conditions that can compromise human health.

## 1 Introduction

**Figure 1:**
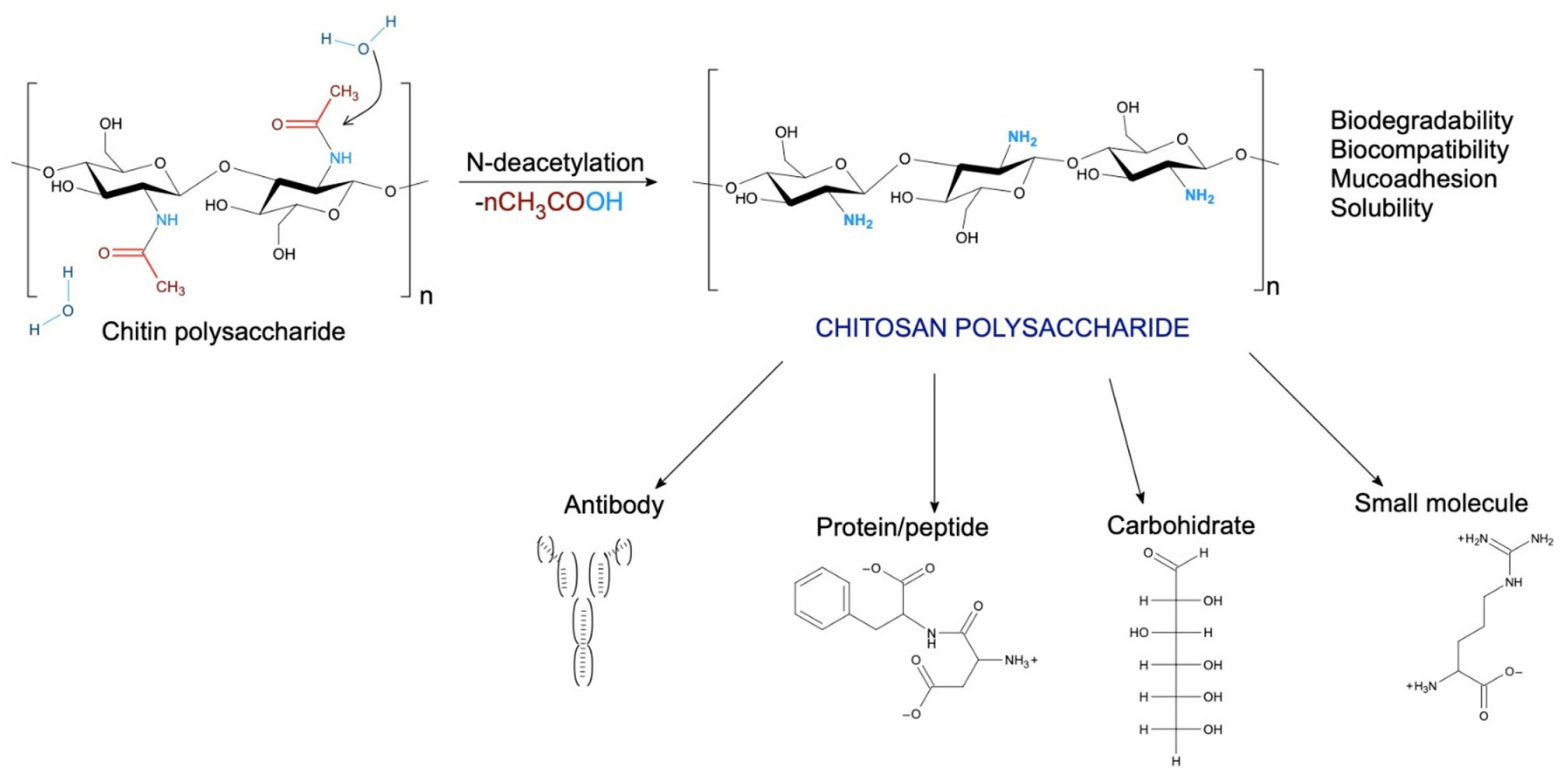
Schematic illustration of *N*-deacetylation of chitin-polysaccharide to chitosan polysaccharide, with different moieties that can target chitosan-based vehicles (based in Mohammadi et al., 2021; Yadav et al., 2022).

Incorporating biopolymers presents promising solutions in the research development of new drug delivery systems. The growing application of biomaterials drives the exploration of novel bioactive, biodegradable, non-toxic, and easy-to-handle polymers for diverse applications. Chitosan (CHS) is a versatile natural copolymer derivate of chitin with mucoadhesive properties, chemically composed of deacetylated ß-(1-4)-linked d-glucosamine and *N*-acetyl-D-glucosamine units (Mohammadi et al., 2021; Yadav et al., 2022). It has been extensively explored for various bio-dental applications due to numerous favorable properties such as biocompatibility, hydrophilicity, biodegradability, and its potential as an antimicrobial or as a support for antibiotic release (Gao et al., 2022). Conventionally, CHS is deacetylated to 85–99% in a random “block-wise” pattern, resulting in a heterogeneous distribution of the remaining acetyl groups (Yadav et al., 2022). CHS in the form of a resorbable membrane can be used to deliver antibiotics such as Chlorhexidine (CHX) to periodontal tissues *in situ* against bacterial and fungal infections (Figure 1) (Mohammadi et al., 2021; Yadav et al., 2022). Antimicrobial resistance is currently a serious problem in oral infections, especially because biofilm formation is known to be a strong contributor. Among the most common bacteria embedded in oral biofilm is *Streptococcus mutans*. These bacteria exhibit multiple resistance mechanisms, for example, their ability to respond to oxidative stress (Tsuda et al., 2002; Nilsson et al., 2019; Gao et al., 2022). Chlorhexidine (CHX) is one of the most widely used antibiotics in treating oral conditions, primarily due to its ability to act as a membrane-active antimicrobial against viable bacteria (Hubbard et al., 2017). For use in the oral cavity, CHS is available and effective in various delivery systems, such as sprays, mouthwashes, glass ionomer cement gels, chips, and varnishes, which are routinely used in the treatment of common oral diseases (Gao et al., 2022).

CHS membranes can be used to design a robust local drug delivery system with the appropriate mechanical and antibacterial properties to maintain intimate contact with the oral tissues (Hemmingsen et al., 2021, 2022; Mohammadi et al., 2021). A usual method for increasing the toughness of the chitosan membrane is by the addition of plasticizers such as Glycerol (G) (Kusmono and Abdurrahim, 2019; Mohammadi et al., 2021; Guo et al., 2024). In addition to mechanical properties, another critical aspect of the oral application of chitosan membranes is their antimicrobial activity (Gao et al., 2022; Guo et al., 2024). In 2018, Podolan successfully synthesized five oral chitosan membranes of different compositions, each containing 5% (m/v) CHS in a 5% acetic acid solution with a range between 1 and 5% (v/v) with CHX gel (0.2, 0.6, 1, and 2%) and a percentage range between 0.5 and 3% of G in three-layer hybrid membrane (Podolan et al., 2018). The antimicrobial test showed good activity against *Candida albicans* and *S. mutans*. Podolan reported that discs containing 0.2, 0.6, and 2% CHX in a three-layer membrane with CHS showed the most significant inhibition zones for *S. mutans*. The 0.2% CHX membrane of chitosan with 5% G showed lower microbial activity compared to the 2.0% CHX gel and membrane, both with and without 5% G. In comparison, other studies have evaluated the effect of G on the mechanical properties of CHS-based membranes with biomedical applications (Cobos et al., 2018; Shojaee Kang Sofla et al., 2020; Stachowiak et al., 2020; Sun et al., 2020; Terukina et al., 2023). Additionally, some studies have reported that the mechanical properties of biopolymer membranes improve with the addition of G (Ren et al., 2018; Harussani et al., 2021; Tarique et al., 2021; Wang et al., 2022; Alasalvar et al., 2023; Xu et al., 2023). These CHS-based membranes with G would establish intimate contact with the tissues and gradually release the antimicrobial agent through the association of CHS with CHX, offering treatment options. In our study, six oral CHS membranes of different compositions were synthesized. They contained 1.0, 3.0, 5.0, 10, 15, and 20% loading of G in a one-layer membrane with CHX at 0.06%. These membranes were successfully synthesized and characterized using FTIR and determining tensile strength, elongation, mass, thickness, rugosity, disintegration, surface pH, swelling capacity, moisture sorption, and water sorption behavior measurements. The main objective of this study is to demonstrate that membranes synthesized from CHS and G can serve as an alternative support for integrating antibiotics or active compounds that can be used in the treatment of oral diseases. In this study, the antimicrobial capacity of CHX against *S. mutans* was used as a model, evaluating the effectiveness of CHS/G/CHX membranes as dispersing agents of an antibiotic such as CHX against *S. mutans*. Specifically, CHS/G/CHX membranes represent a promising alternative for treating diseases caused by *S. mutans*, such as caries and periodontal diseases.

## 2 Materials and methods

High molecular weight CHS in powder form with a deacetylation degree more significant than 85% was obtained from Sigma-Aldrich (USA). Glacial Acetic acid and distilled water were acquired from Sigma Aldrich (USA). Glycerol spectrophotometric grade, 99.5%+, was obtained from Acros, New Jersey, USA. Chlorhexidine gluconate solution 2% (Genhexis RC, ZEYCO) simulated saliva (pH 7.38) was obtained from Viarden Lab. *S. mutans*, CDBB-B-1455, was obtained from CINVESTAV-IPN.

### 2.1 Preparation of chitosan suspension

To prepare 400 mg of chitosan powder, 40 mL of deionized water was added to a flask and magnetically stirred at 60°C. Subsequently, an aqueous solution of acetic acid (2%, v/v) was added to the mixture, and stirring continued for 15 min until complete dissolution of the CHS, resulting in a 2.0% CHS solution. Simultaneously, to demonstrate the impact of the plasticizer level on the membrane properties, varying concentrations of G (1, 3, 5, 10, 15, and 20 % v/v) were added to the CHS solution and stirring continued for 15 min until the CHS solution transformed into a flocculent suspension.

### 2.2 Preparation of chitosan membranes

The CHS membranes were prepared using a modified casting and solvent evaporation method (Korelc et al., 2023). Twenty milliliters of the CHS suspension prepared using the procedure described above were cast onto Petri dishes (diameter = 16 cm) using an analytical scale. The prepared plates were dried at ambient conditions for 72 h until the water was evaporated entirely. The appropriate plasticizer level was identified empirically by the preparation of membranes with different G concentrations. Then membranes were observed for suitable flexibility (absence of breaking or deformation during handling as well as the ability to return to the initial stage without sticking to the contact dental surface). The final sample designation and amounts of CHS/G used for each sample are shown in Table 1. The chitosan/glycerol/chlorhexidine (CHS/G/CHX) membrane was prepared at the same formulation, with 1.2 mL of CHX gluconate at 2% to produce formulations at 1, 3, 5, and 10% of loading G.

**Table 1:** Sample designation and composition in terms of Chitosan (CHS), Glycerol (G), and Chlorhexidine (CHX) content.

**Sample**	Parts (%v/v)^*^
CHS/G1	99/1
CHS/G3	97/3
CHS/G5	95/5
CHS/G10	90/10
CHS/G15	85/15
CHS/G20	80/20
CHS/G1/CHX	99/0.06/1
CHS/G3/CHX	97/0.06/3
CHS/G5/CHX	95/0.06/5
CHS/G10/CHX	90/0.06/10
CHS/G15/CHX	85/0.06/15
CHS/G20/CHX	80/0.06/20

^*^Percentage of G and CHX are based on the CHS solution.

### 2.3 *In vitro* characterization

#### 2.3.1 Visual inspection and content uniformity

The membrane samples were analyzed with an optical microscopy LEYCA DM-1000 at 4X and 10X amplification to identify the presence of some imperfections, both after preparation and during storage. The uniformity of CHX and G content was assessed by cutting discs of 0.6 cm in diameter from five different areas of the studied membrane samples.

### 2.4 Scanning electron microscopy analysis

The surface images of the membranes were observed by scanning electron microscope (SEM, JEOL, JSM-6610 LV, Japan). These membranes were dried at 75°C for 48 h, and then dried fragments were broken and coated with gold by sputtering to produce electric conductivity. SEM images of the membrane surface were taken in a low vacuum condition operating at 5 kV.

### 2.5 Fourier transform infrared (FTIR-ATR) spectra analysis

For Fourier transform infrared spectroscopy (FTIR Perkin-Elmer, spectrum 2000), the spectra of the samples of chitosan film and its glycerol films were obtained with 32 scans in the interval between 400 and 4,000 cm^−1^, with a resolution of 4 cm^−1^. The spectra were obtained using the ATR (attenuated total reflectance) accessory (Bruker, Vertex 70 model).

### 2.6 Tensile strength

The tensile tests were performed according to the method described in the literature (Kusmono and Abdurrahim, 2019) using a universal testing machine (Instron 5567, Instron, Norwood, MA, USA) at 5 mm/min crosshead speed. All samples were cut to the standard shape of 20 mm wide and 150 mm gauge length. The measurement was performed in the ambient condition (25°C, relative humidity of 48 ± 2%). Tensile strength and elongation at break were evaluated (*n* = 5), and the average values were reported for accuracy.

### 2.7 Mass and thickness of membranes

Depending on the G ratio, membranes were weighed separately using the analytical balance (OHAUS Adventure Pro AV265C: 260 g (SD ± 0.1 mg) (l: ± 0.3 mg), after 48 h stored at room temperature. The mean mass and standard deviation (SD) were calculated. The thickness of the membranes was measured using a micrometer screw (SHAHE-Model T152002FR, 0–25 ± 0.04 mm) in the center and each corner, taking an average of the five values per unit.

### 2.8 Surface pH

A disc unit (*n* = 3) was placed in a flask containing 5 mL of artificial saliva solution. The formulation was allowed to swell for 5 min and was subsequently removed from the flask (Korelc et al., 2023). The resulting pH of the liquid was recorded using a pH meter (ROCA-Model PHS-3CU) at room temperature and was taken as an indication of the formulations' surface pH.

### 2.9 Moisture sorption

The membranes' moisture sorption was studied by exposing them to 75% relative humidity (RH) using a desiccator with an oversaturated NaCl solution at room temperature. The units were pre-weighed (m_1_) and stored in a humid desiccator for 10 days. Afterwards, they were re-weighed (m_2_). Moisture sorption was calculated in the same manner as Swelling capacity and multiplied by 100 to obtain the sorption percentage (Korelc et al., 2023). The experiment was performed in triplicate.

### 2.10 Roughness

The membrane surface roughness parameters were measured with a previous roughness meter (Mitutoyo SJ-301, Mitutoyo American Corporation, EU), working at a speed of 0.25 mm/s and a cutoff distance of 0.8 × 5 mm (Ładniak et al., 2021). The membranes were fixed with double-sided tape parallel to the tip of the roughness gauge. Five membranes were used (*n* = 5).

### 2.11 Disintegration

The disintegration test was performed using a modified disc method from literature (Korelc et al., 2023). A membrane disc was immersed into a flask containing 5 mL of artificial saliva solution (pH = 6.80) and shaken at 60 rpm at 36°C to imitate the movements in the oral cavity. During the experiment, the disintegration time for separate units of membrane discs was observed. In cases where a coherent matrix remained after 24 h, the sample was recorded as not disintegrated. Three parallels were tested for each formulation.

### 2.12 Water sorption behavior

CHS membranes were cut into small discs (0.6 cm diameter), desiccated overnight under vacuum, and weighed to determine their dry mass. The weighed discs were placed in a flask containing 5 mL of artificial saliva solution pH 7.38 at 36°C (Korelc et al., 2023). The swelling kinetics were evaluated by periodically measuring the weight increment of the samples with respect to dry membranes using an analytical scale with a precision of 0.001 g. After gently bottling the surface with a tissue to remove the excess artificial saliva solution, the samples were weighed, and the respective values were recorded until 24 h. The water gain (WG) was calculated as Equation 1:


(1) $$\begin{array}{c} W.G.\;\left(\%\right)=\frac{m2-m1}{m1}x\;100 \end{array}$$

where *m*2 = *m*_eetfilm_ and *m*1 = _dryfilm_ are the weights of the wet and dry membranes, respectively.

### 2.13 Swelling capacity

The swelling capacity of membranes was evaluated using a modified test from literature (Korelc et al., 2023), where a membrane with a determined mass (*m*_1_) was placed in a glass flask and immersed in 5 mL of artificial saliva solution (pH = 7.38) at 37°C, to mimic the temperature conditions in the oral cavity. The membrane was allowed to swell for 5 min. Its mass (*m*_2_) was recorded after gently wiping the product with a piece of tissue paper to remove the surface water. The swelling index represents the mass gained with respect to the mass of a dehydrated membrane and was calculated according to Equation 2. The experiment was carried out in triplicates.


(2) $$\begin{array}{c} S.I.\;=\frac{m2-m1}{m1} \end{array}$$

### 2.14 Antibacterial analysis

*S. mutans* (CDBB-B-1455) cells were grown in an overnight liquid culture with brain heart infusion (BHI) medium, which is composed of a mixture of calf brain infusion and bovine heart infusion, peptone, NaCl, glucose, and disodium phosphate. Bacteria were grown for 24 h without shaking and under anaerobic conditions at a temperature of 37°C and a CO_2_ concentration of 5% using the ESCO Cell Culture CO_2_ INCUBATOR. After 24 h, the optical density of the culture was measured by taking 100 μL of sample. To perform the inhibition tests, 100 μL of the cell culture was spread evenly onto a plate containing a solid BHI medium. The membranes, which had different concentrations of G and were approximately 5 millimeters in size and round, were sterilized in a class 2 biosafety cabinet (LABCONCO et al.) using UV light for 20 min on each side. The sterile membranes were positioned at the center of the plates where *S. mutans* had been spread, employing a technique akin to sensitized tests. Subsequently, they were incubated for 24 h under the same anaerobic conditions mentioned earlier. Following this incubation period, the inhibition halos formed were measured using a digital vernier (GENERAL^®^ ULTRATECH^TM^). In each replicate, the number of colony-forming units per milliliter (CFU/mL) was determined using the “Massive Stamping Drop Plate” (MSDP) method (Corral-Lugo et al., 2012).

### 2.15 Statistical analysis

All values are presented as the mean ± SD (standard deviation). Statistical analyses were performed using Sigma 2.7, 2016 TYEvolution, USA to evaluate the data obtained. One-way analysis of variance (ANOVA) was used to assess statistically significant differences in three or more groups. Additionally, Tukey's multiple comparisons test was performed. *p* < 0.05 was accepted as statistically significant.

## 3 Results and discussion

### 3.1 Physical appearance of CHS/G/CHX membranes

**Figure 2:**
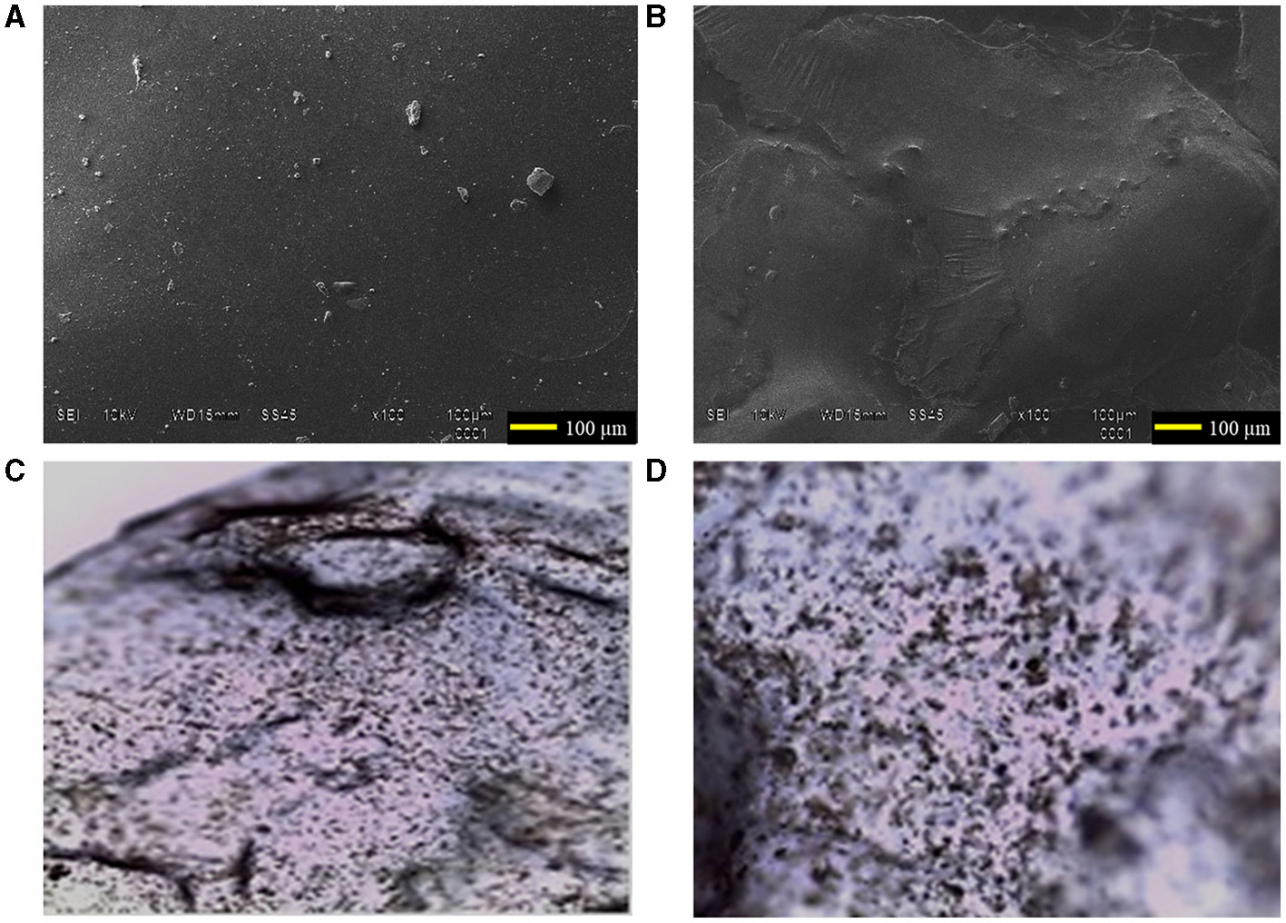
Scanning electron microscope images of the surface of CHS/G/CHX membranes at 100 magnifications: **(A)** CHS/G/CHX membrane at 1% of G. **(B)** CHS/G/CHX membrane at 3% of G. Optical images of the surface CHS/G/CHX membrane at 3% of G **(C)** 4X and **(D)** 10X after 5 min in simulated saliva solution.

The CHS/CHX loaded membranes with G at 1 and 3% presented a homogeneous appearance, colorless, with no evidence of drug separation upon visual inspection (Figure 2). However, CHS/CHX loaded membranes and CHS membranes with G at 15 and 20% exhibited a non-homogeneous, gummy, and very elastic appearance. After 2 months, physical changes such as color, texture, and other physical parameters were observed. The concentration of chlorhexidine present in the CHS/G/CHX membranes was 0.06%. Clinical studies have shown that mouthwashes containing alcohol and concentrations higher than 0.2% of CHX can lead to significant extrinsic dental pigmentation, whereas no dental pigmentation has been observed with alcohol-free mouthwashes (Polizzi et al., 2020). Based on these studies, it is proposed that by using a lower concentration of CHX (half of that used in mouthwashes), the likelihood of dental pigmentation or damage occurring would be minimal or non-existent. It is important to highlight that the frequent use of chlorhexidine as an antiseptic in oral hygiene or after periodontal surgery is considered a potential factor in the emergence of cross-resistance to antibiotics. Studies have shown that treatment with CHX could promote microorganisms associated with caries, potentially allowing the expression of tetracycline resistance genes (Bartsch et al., 2024). Considering this information, it is relevant to emphasize that unlike mouthwash, which comes into contact with all dental surfaces, CHS/G/CHX membranes are designed for application to specific sites, thereby avoiding contact with healthy dental tissues.

### 3.2 Scanning electron microscopy analysis

The top surface of the CHS/G/CHX membranes prepared with 1 and 3% of G was examined using scanning electron microscopy (SEM) after incubating at 37°C in the oven for 72 h to investigate the influence of G concentration on the morphology of the resulting membrane. The surface morphology is a widely used technique to study the sub-microscopic details of different drug delivery systems, including membranes. Figure 2 shows the top surface of CHS/G/CHX membranes with the absence and presence of few agglomerations of chitosan matrix obtained by this synthesis method. The SEM image of the surface morphology of CHS/G/CHX at 1% (Figure 2A) and CHS/G/CHX at 3% (Figure 2B) membranes suggests that all formulation constituents were mixed and uniformly distributed in the membrane with the presence of some drug particles on the surface. Similar results were presented in other studies with doxycycline mucoadhesive buccal carbohydrate polymer-based membranes with G by Dinte et al. (2023). An increase in G content resulted in the formation of stretch marks on the top surface of the membrane and a more elastic appearance. Similar observations were achieved by other studies (Caicedo et al., 2022). No physical changes, such as color, were observed during storage for 2 months. Figures 2C, D show optical images at 4X and 10X magnification, respectively, captured in a simulated saliva solution at 37°C after 5 min, observing the CHS/G/CHX at 3% membrane surface appearing compact and smooth.

### 3.3 FTIR-ATR analysis

**Figure 3:**
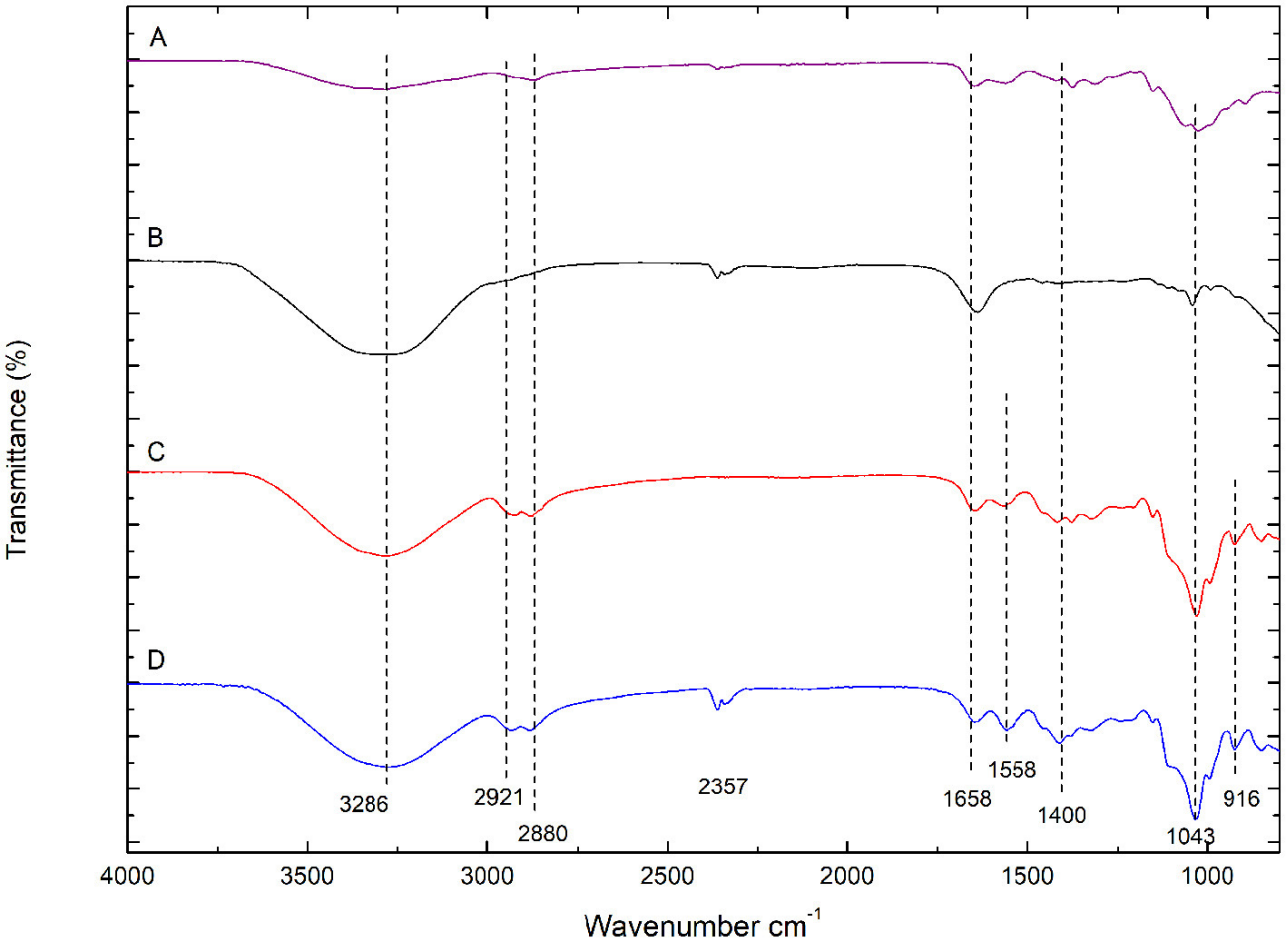
FTIR-ATR spectra of **(A)** CHS powder (purple), **(B)** CHX gluconate (black), **(C)** CHS/G 1% membrane (red), **(D)** CHS/G/CHX 1% (blue) membranes.

Figure 3 presents the FTIR-ATR spectra of CHS powder (purple), CHX (black), CHS/G membrane (red), and CHS/G membrane containing CHX (blue). These spectra indicate that the intercalated structure of G and CHX are found in this CHS membrane (blue). The absorption peaks at 3,286 cm^−1^ correspond to the stretching vibration of N-H, while peaks at 2,921 and 2,280 cm^−1^ are assigned to the typical C-H stretching vibration in -CH_2_ and -CH_3_ of CHS, respectively. In addition, the peak at 1,658 cm^−1^ corresponds to C=O stretching (amide I), and the peak at 1,568 cm^−1^ is assigned to N-H bending (amide II). The peak at 1,400 cm^−1^ is assigned to C-N stretching (amide II), and the peak at 1,043 cm^−1^ is assigned to the OH-O bond of G with CHS. In the spectra of the chitosan membrane with G (red and blue), the peak at 1,043 cm^−1^ is attributed to the hydrogen bond O-H-O vibration of G, indicating the presence of G in the CHS membranes. The intensity of the peak at 1,031 cm^−1^ is not observed in the spectra of powdered CHS (purple) and CHX (purple).

**Figure 4:**
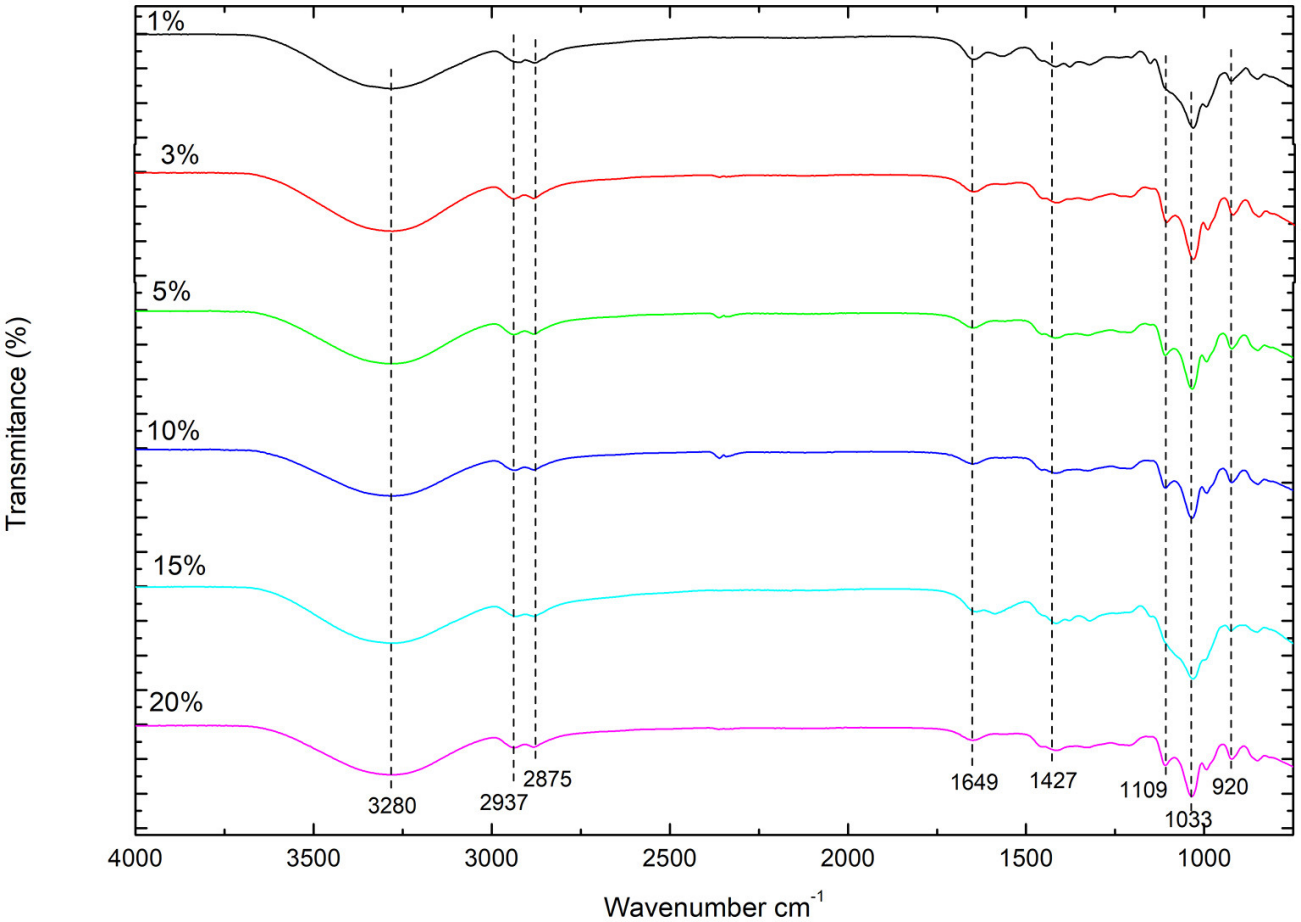
FTIR spectra of CHS/G/CHX at ratio G 1, 3, 5, 10, 15, and 20%.

The characteristic peak of CHX can be observed in the CHS/G/CHX membrane. The peaks between 3,400 and 3,200 cm^−1^ correspond to the stretching vibration of N-H, OH groups of gluconates, and OH groups from absorbed water. A band also appears at 1,640 cm^−1^, which could be related to the C=N (imine) stretching bond of the biguanide group. While Figure 4 shows that CHS/G loaded membranes at 1, 3, 5, 15, and 20% presented no difference in FTIR spectra. The G-plasticized CHS membrane presents similar spectral bands to those observed in the CHS powder, purple FTIR-spectra (Figure 4). However, the presence of G affects the position and intensity of the NH${\;}_{3}^{+}$ band of the unplasticized CHS membrane. That band shifts to reduced order and higher frequency to 1,558 cm^−1^ from CHS/G 1% of G membrane (red spectra) to CHS/G/CHX 1% of G membranes (blue spectra) (Figure 3). Similar results are observed by Cobos et al. (2018).

### 3.4 Tensile properties

The mechanical properties of the membranes were a major concern for the practical application. Elongation and tensile strength studies are very effective in evaluating the mechanical endurance of membranes. The testing results of mechanical stability including tensile strength and elongation at break values are listed in Table 2.

**Table 2:** Overview of characteristics of membrane of CHS/G and CHS/G/CHX at various loading of G, including tensile strength (MPa) and elongation at break (%) from stress strain test at 5 mm/min speed (*n* = 5), mass (mg) (*n* = 5), thickness (μm) (*n* = 5), surface pH (*n* = 3), and moisture sorption (%) after 10 days (*n* = 3) (values are given as mean ± SD).

**Membrane composition**	**Tensile strength (MPa ±SD)**	**Elongation at break (%)**	**Thickness (μm)**	**Mass (mg)**	**Surface pH**	**Moisture sorption (%)**
CHS/G1	0.696 ± 0.34a	30.50 ± 10.99	155.6 ± 45.6	46.9 ± 5.5a	6.72 ± 0.006	21.35 ± 5.87a
CHS/G3	0.193 ± 0.15	95.43 ± 24.95	249.0 ± 70.0a	99.9 ± 4.7b	8.81 ± 0.065	37.00 ± 3.38b
CHS/G5	0.164 ± 0.10	127.73 ± 1.59	182.8 ± 27.0b	1,455 ± 86.4	7.45 ± 0.010	41.63 ± 1.13c
CHS/G10	0.161 ± 0.11	141.33 ± 75.54a	284.0 ± 36.0	2,707 ± 135.2c	7.04± 0.066	43.89 ± 1.08d
CHS/G15	0.065 ± 0.01b	159.15 ± 72.69b	665.0 ± 32.0c	3,779 ± 129.5d	6.35 ± 0.182	45.40 ± 3.18e
CHS/G20	0.098 ± 0.30c	130.28 ± 3.25c	359.0 ± 115	5,444 ± 929.6	6.63 ± 0.010	41.12 ± 1.11f
CHS/G1/CHX	0.322 ± 0.06a	32.25 ± 3.73	108.4 ± 27.2	560.8 ± 56.5a	6.62 ± 0.096	1.40 ± 0.73a
CHS/G3/CHX	0.458 ± 0.16	100.35 ± 13.61	145.4 ± 42.4a	998.6 ± 299.3b	7.7 ± 0.041	7.01 ± 3.82b
CHS/G5/CHX	0.135 ± 0.08	132.42 ± 7.60	297.0 ± 92.3b	1,421.4 ± 156.5	7.01 ± 0.081	15.01 ± 4.84c
CHS/G10/CHX	0.144 ± 0.12	203.48 ± 8.50a	214.2 ± 44.9	3,097.6 ± 185.7c	7.26 ± 0.229	25.87 ± 1.60d
CHS/G15/CHX	0.038 ± 0.012b	223.08 ± 11.52b	461.8 ± 77.1c	4,383.8 ± 125.6d	6.12 ± 0.466	30.00 ± 0.75e
CHS/G20/CHX	0.054 ± 0.03c	216.58 ± 14.58c	557.60 ± 63.1	5,844.2 ± 314.4	6.39 ± 0.006	34.33 ± 0.53f

Values with same letters in the same column indicate are significantly different (Tukey test, *p* < 0.05).

**Figure 5:**
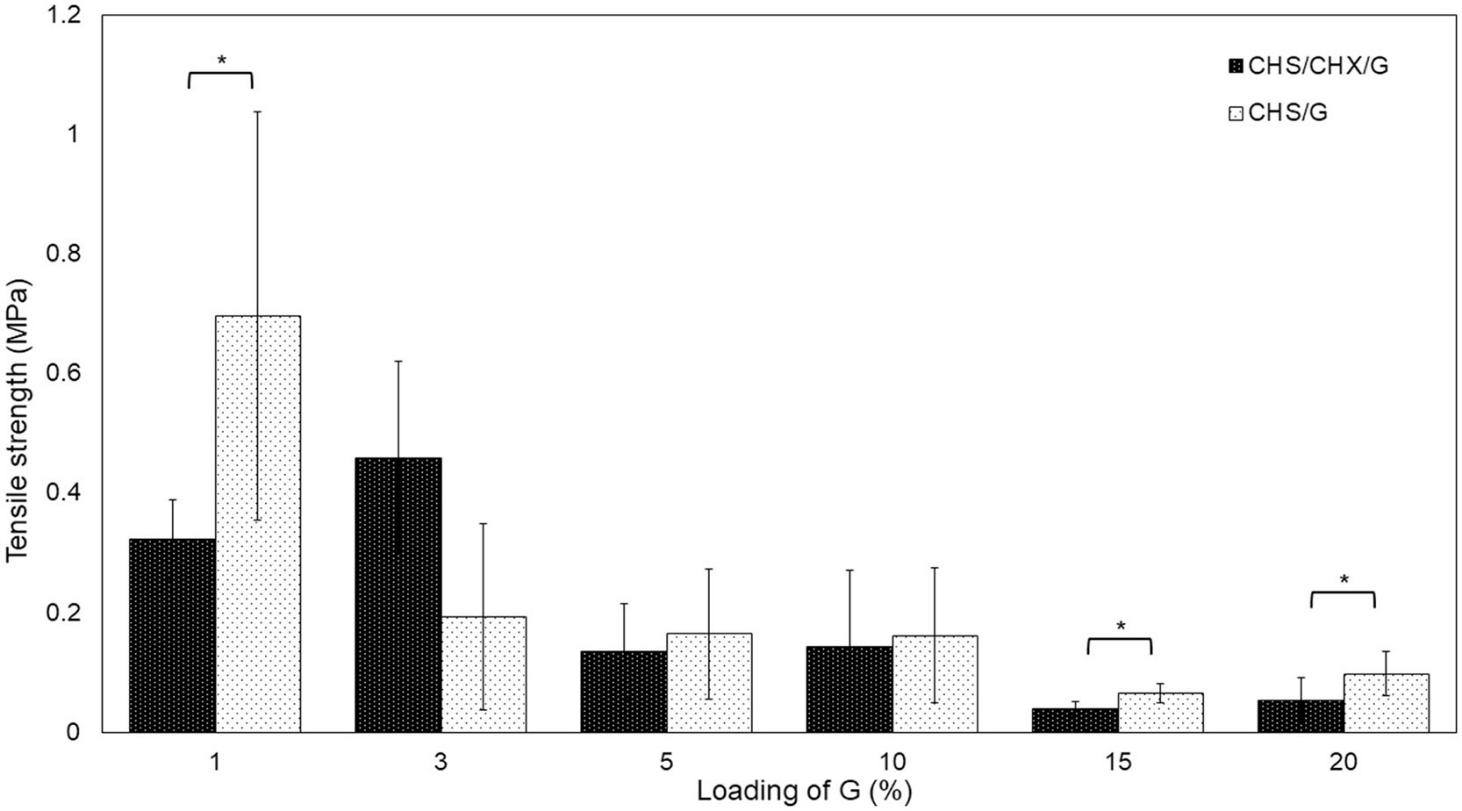
Effect of loading G on tensile strength of CHS/G and CHS/G/CHX membranes obtained from stress-strain test, *n* = 5 (mean ± SD). Vertical bars indicate standard error of means. Asterisk symbols indicate a significant differences (Tukey test, *p* < 0.05).

Tensile strength is an important indicator that characterizes membranes and their ability to withstand loads. An increase in this parameter is required for oral membranes (Dinte et al., 2023). In Figure 5, the effect of G loading on the tensile strength of the CHS membrane is shown. The addition of 1–3% of G increased the tensile strength of the CHS/CHX membrane in the range of 0.322 ± 0.06–0.458 ± 0.16 MPa but decreased with further G addition of 5–20% in a range of 0.135 ± 0.08–0.038 ± 0.01 MPa, a lower value being observed at CHS/G15/CHX. In agreement with our findings, previous studies reported that when using increased plasticizer as G in CHS-based membranes (Cobos et al., 2018), the tensile strength decreased, and with other biopolymers membranes (Ren et al., 2018; Shojaee Kang Sofla et al., 2020; Harussani et al., 2021; Tarique et al., 2021; Caicedo et al., 2022; Wang et al., 2022; Alasalvar et al., 2023; Terukina et al., 2023). In the present study, the highest value in tensile strength was obtained at the CHS/G3/CHX and CHS/G1, 0.458 ± 0.16 and 0.696 ± 0.34 MPa, respectively. Tensile strength values present CHS/G membranes decrease with addition of G at 1–20%, but the difference is statistically significant compared between CHS/G1–CHS/G1/CHX, CHS/G15–CHS/G15/CHX, and CHS/G20 – CHS/G20/CHX membranes, *p* < 0.05. The values obtained are lowest to those obtained in studies with the use of G (Kusmono and Abdurrahim, 2019) but highest compared with glycerin as plasticizer in oral chitosan-based membranes (Arpa et al., 2023) or hydroxypropylmethyl cellulose-based membranes (Dinte et al., 2023).

**Figure 6:**
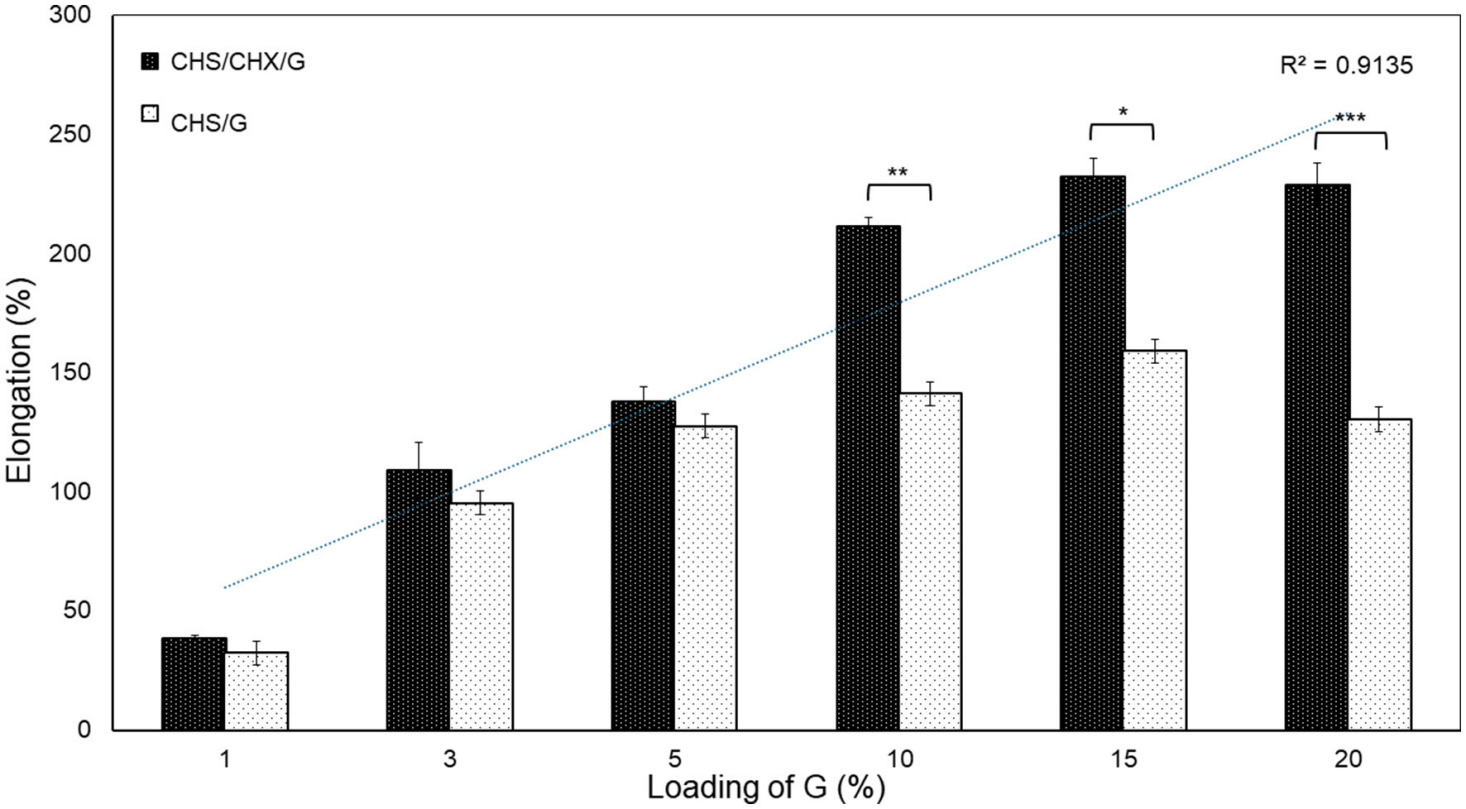
Effect of loading G on elongation at break of CHS/G and CHS/G/CHX membranes obtained from stress-strain test, *n* = 5 (mean ± SD). Vertical bars indicate standard error of means. Asterisk symbols significant differences (Tukey test, *p* < 0.05).

As represented in Figure 6, the flexibility of membranes increased with loading G. The studied membranes presented elongation values in the range of 32.55 ± 14.49%−159.15 ± 72.69% to CHS/G membranes, while elongation values in the range of 32.25 ± 3.73%−216.58 ± 14.58% to CHS/G/CHX membranes (Table 2). The elongation values obtained are comparable to those obtained in previous studies of CHS membranes (Cobos et al., 2018; Kusmono and Abdurrahim, 2019; Arpa et al., 2023) and with other biopolymers (Shojaee Kang Sofla et al., 2020; Harussani et al., 2021; Caicedo et al., 2022; Wang et al., 2022; Alasalvar et al., 2023). As show in Figure 6, the differences between the elongation values of CHS/G and CHS/G/CHX membranes were statistically significant (CHS/G10–CHS/G10/CHX, *p* < 0.008; CHS/G15–CHS/G15/CHX, *p* < 0.05; CHS/G20–CHS/G20/CHX, *p* < 0.000).

The enhancement in tensile strength of the CHS/G membrane may be mainly attributed to the homogeneous dispersion of high rigid G molecules with a high aspect ratio in the copolymer matrix of CHS, subsequently resulting in hydrogen bonding between the -OH and -NH_2_ deacetylate of CHS and -OH groups of G. On the other hand, the tensile strength was drastically reduced by the presence of G more than 5%. The addition of G reduced the tensile strength of CHS/CHX membrane. Some membranes, CHS/G/CHX 15 and 20%, were agglomerates after force by induced local stress; concentration G with CHX were believed to be responsible for the decrease in tensile strength.

### 3.5 Mass and thickness of the membrane

The thickness values were in the range of 155.6 ± 45.6–665.0 ± 32.0 μm to the CHS/G membrane and thickness values ranged from 108.4 ± 27.2 to 557.60 ± 63.1 μm to CHS/G/CHX membrane (Table 2). The thickness increased in order CHS/G1 < CHS/G3 < CHS/G5 < CHS/G10 < CHS/G15, the membrane with greatest thickness being CHS/G15, which has 15% loading G and the difference is statistically significant compared to the other four membranes (CHS/G1–CHS/G15, CHS/G3–CHS/G15, CHS/G5–CHS/G15, CHS/G10–CHS/G15, *p* < 0.05) (Table 2). This change in thickness caused by increase in the content of G was similar to that reported with CHS in several studies (Tarique et al., 2021) and other biopolymers membranes (Caicedo et al., 2022; Wang et al., 2022; Alasalvar et al., 2023). In contrast, the thickness values to CHS/G20 membrane decreased to 359.0 ± 115 μm which indicates that loading G at 20% interferes the copolymer matrix. Similar results were reported by Harussani with cornstarch biopolymer membranes; the thickness decreased at the highest concentrations of G, 30, 45, and 60% (Harussani et al., 2021). Thus, the difference in compared thickness between membranes is statistically significant to CHS/G3–CHS/G3/CHX, *p* < 0.02; CHS/G5–CHS/G5/CHX, *p* < 0.03; and CHS/G15–CHS/G15/CHX, *p* < 0.003.

**Figure 7:**
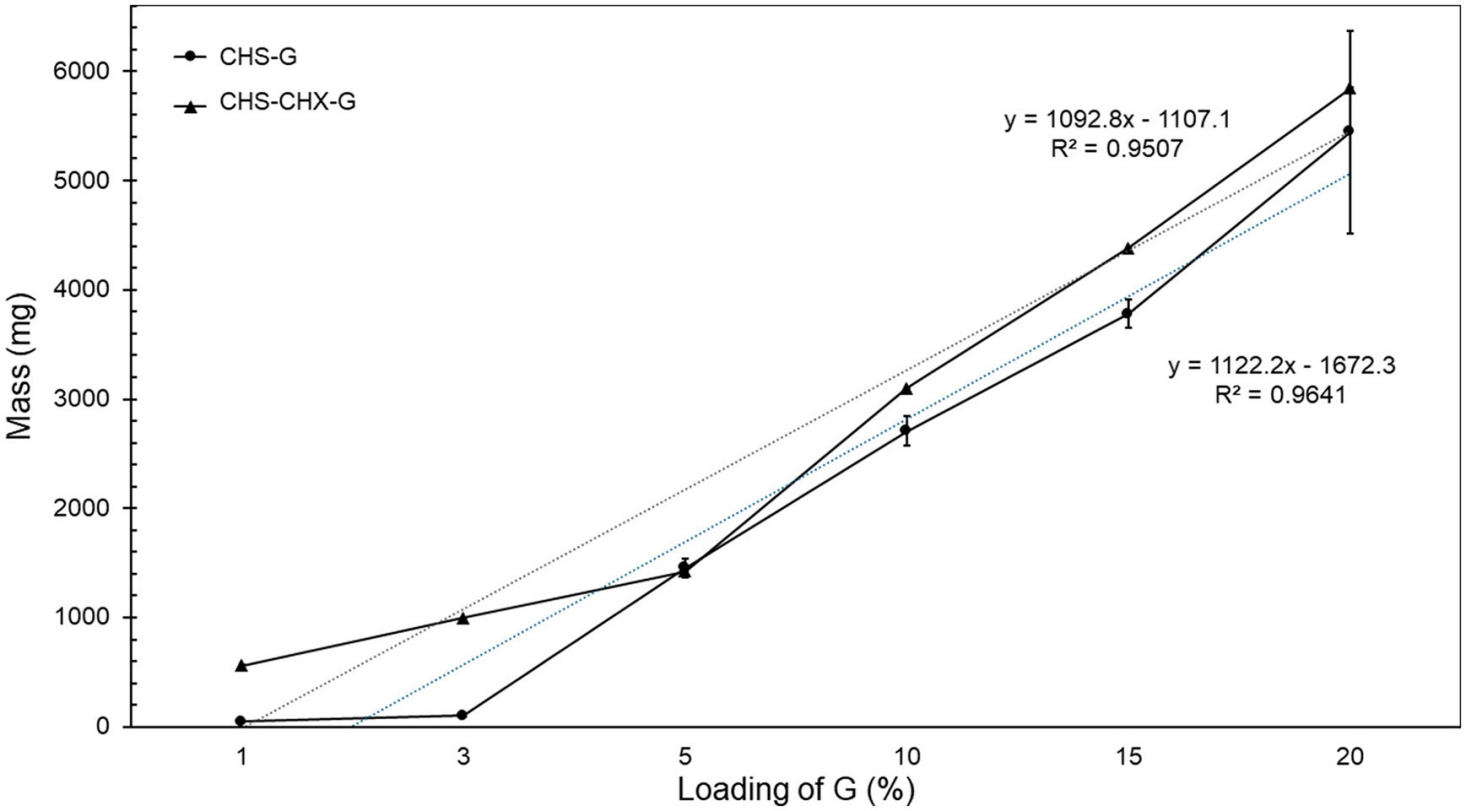
Effect of loading G on mass (mg) of CHS/G and CHS/G/CHX membranes, after 48 h at room temperature, *n* = 5 (mean ± SD).

The observed mass of membranes, as shown in Table 2, demonstrated a direct proportionality with the increase in G content in the formulation. The membranes' mass showed a linear relationship [*R*^2^ = 0.9507 (CHS/G/CHX) and 0.9641 (CHS/G) (Figure 7)]. The mass values were in the range of 560.8 ± 56.5–5,844.2 ± 314.4 mg to CHS/G/CHX membranes, and range of 46.9 ± 5.5–5,444 ± 929.6 mg to CHS/G membranes. The mass values increased in order CHS/G1 < CHS/G3 < CHS/G5 < CHS/G10 < CHS/G15, the membrane with the greatest mass being CHS/G15, similar results with the thickness values in CHS/G20, the mass decreased at 359.0 ± 115 mg for CHS/G20 membrane. In contrast, the mass values increased to CHS/G1/CHX < CHS/G3/CHX < CHS/G10/CHX < CHS/G15/CHX < CHS/G20/CHX. Differences between membranes' mass values were statistically significant (CHS/G1–CHS/CHX/1, *p* < 0.000; CHS/G3–CHS/G3CHX, *p* < 0.000; CHS/G10–CHS/G10/CHX, *p* < 0.005; CHS/G15–CHS/G15/CHX, *p* < 0.000). Based on these mass values, we observed an increase in mass with higher G loading in the CHS membrane as show in Figure 7.

### 3.6 Surface pH

The pH measurement provided information on the tolerance of the membranes in the oral cavity because acidic or basic pH can irritate the oral mucosa. The studied membrane samples present values between 6.35 ± 0.182, a lower value being observed at CHS/G 15, and 8.81 ± 0.065 to CHS/G membrane and pH range of 6.12 ± 0.466–7.7 ± 0.041 (Table 2) to CHS/G/CHX membranes, a lower value being observed at CHS/G15/CHX. A pH range of 5.5–7.0 is considered tolerated (Dinte et al., 2023), but other authors considered that pH < 4.0 or pH > 8 may result in irritation of oral mucosa (Kusmono and Abdurrahim, 2019). In this respect, membranes containing CHS/G and CHS/G/CHX are not considered excessively acidic or alkaline, as they provide pH values within an acceptable range to avoid irritation of the oral mucosa. The pH values following 7.0 showed CHS/G/CHX membranes at 3, 5, and 10%, as shown in Table 2.

### 3.7 Moisture sorption

**Figure 8:**
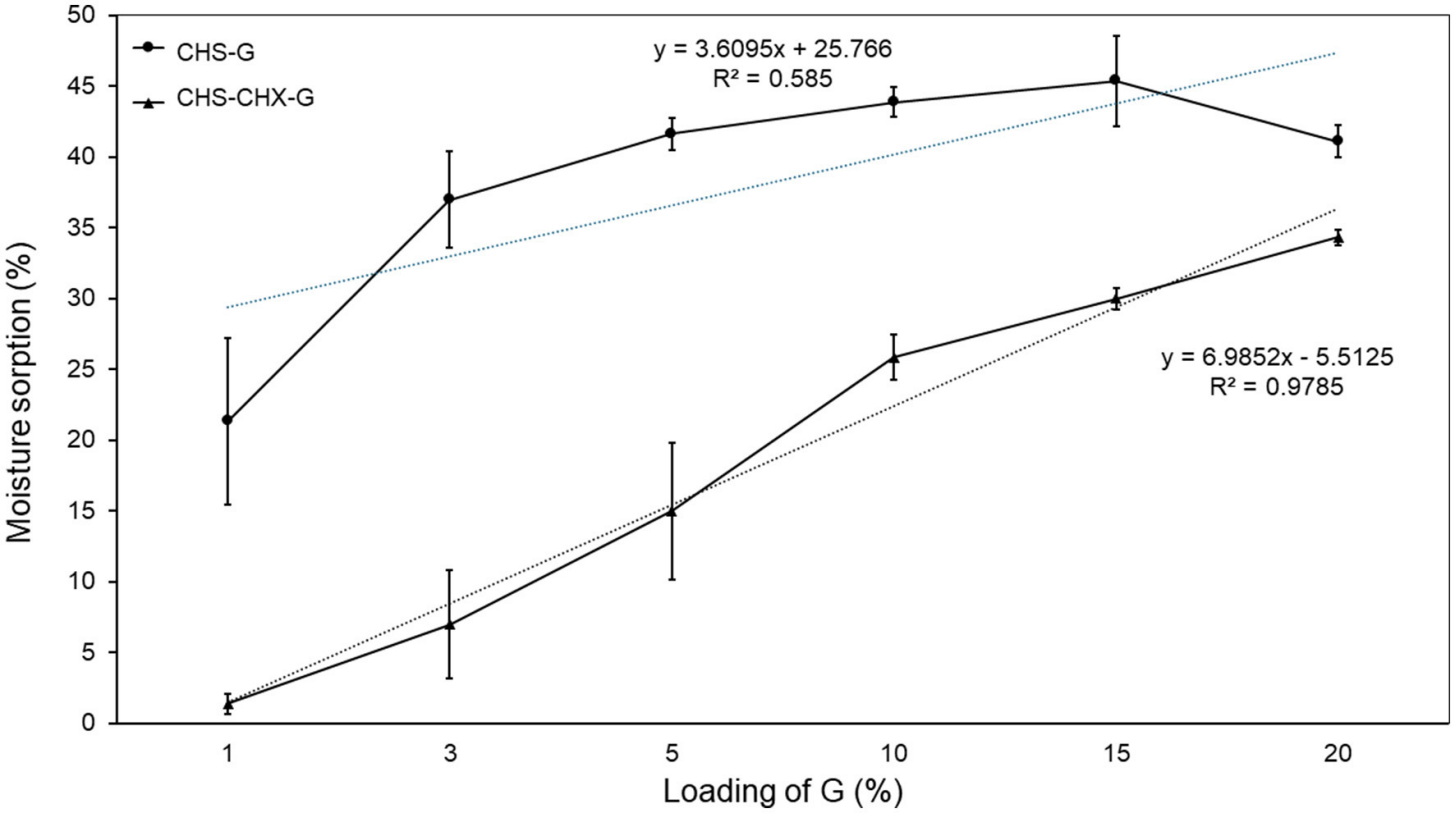
Effect of loading G on the moisture sorption (%) of CHS/G and CHS/G/CHX membranes after 10 days in saturated solution saline at room temperature, *n* = 3 (mean ± SD).

Moisture sorption studies provide insight into the water-holding capabilities of the polymer and plasticizer used in membrane preparation. The moisture absorption of the membranes was found to be between 21.35 ± 5.87% and 45.40 ± 3.18% to CHS/G membranes, a lower value being observed at CHS/G1 and a range of 1.40 ± 0.73%−34.33 ± 0.53% to CHS/G/CHX membranes, with a lower value being observed at CHS/G1/CHX (Table 2). The moisture sorption values showed a linear relationship [*R*^2^ = 0.9785 (CHS/G/CHX) and *R*^2^ = 0.585 (CHS/G) (Figure 8)]. In formulation pairs containing the same amount of G, membranes have statistically significant differences (Tukey test, *p* < 0.05) (Table 2). Previous studies showed that with an increasing plasticizer concentration, there was increased moisture sorption (Tarique et al., 2021; Arpa et al., 2023; Xu et al., 2023). When it reached loading G, similar results were observed with other biopolymer membranes, such as cornstarch-based membranes (Harussani et al., 2021; Tarique et al., 2021; Wang et al., 2022). This result was associated with the hygroscopic property of G. Our experiment results revealed that the CHS/G membrane gained more water than the CHS/G/CHX membrane at the same loading G for 10 days in saline solution at room temperature.

### 3.8 Roughness

Rugosity is one of the most useful parameters for assessing the overall appearance of the membrane's surface. In this investigation, we analyzed the effects of G loading on membrane surface roughness. The Ra and Rz values of CHS/G and CHS/G/CHX membranes are listed in Table 3. The Ra values were in the range of 2.458 ± 1.64–4.300 ± 3.15 μm to the CHS/G membrane. Ra values ranged from 1.124 ± 0.45 to 5.038 ± 0.47 μm to CHS/G/CHX membrane. The Rz values were in the range of 10.23 ± 5.82–22.60 ± 0.49 μm to the CHS/G membrane. The Rz values were in the range of 6.97 ± 1.88–27.25 ± 3.34 μm to the CHS/G/CHX membrane. Based on the results presented in Table 3, we can conclude that the surface roughness parameters of the studied membranes increase with higher G loading. Han et al. (2013) reported similar results with polyethylene glycol as an additive in cellulose acetate/carboxymethyl cellulose membranes. The increasing roughness of the membranes was consistent with the top surface images revealed by scanning electron microscopy (Figure 2A) and optical image (Figure 2C).

**Table 3:** Effect of G loading on surface parameters (μm) of the CHS/G membranes and CHS/G/CHX membranes (*n* = 5) and swelling index (Si) at 5 min (*n* = 3) (values are given as mean ± SD).

**Membrane**	**Surface roughness data**		**Si**
	**Ra (**μ**m)**	**Rz (**μ**m)**	
CHS/G1	3.870 ± 1.87	18.82 ± 8.45	4.86 ± 1.40a
CHS/G3	4.300 ± 3.15	16.49 ± 9.30	7.85 ±1.07b
CHS/G5	2.458 ± 1.64	10.23 ± 5.82	0.28 ± 0.10c
CHS/G10	2.810 ± 0.98	13.20 ± 4.64	0.06 ± 0.01d
CHS/G15	4.163 ± 0.53	22.60 ± 0.49	–
CHS/G20	4.287 ± 1.81	20.35 ± 7.11	–
CHS/G1/CHX	3.028 ± 1.00	13.18 ± 3.29	8.07 ± 0.58a
CHS/G3/CHX	1.620 ± 0.72	6.97 ± 1.88	1.82 ± 0.02b
CHS/G5/CHX	1.124 ± 0.45	7.04 ± 2.67	2.04 ± 0.21c
CHS/G10/CHX	5.038 ± 0.47	27.25 ± 3.34	0.78 ± 0.32d
CHS/G15/CHX	4.086 ± 0.79	17.38 ± 4.76	–
CHS/G20/CHX	2.430 ± 1.04	9.70 ± 3.47	–

(–) indicate disintegration before 5 min. Values with same letters in the same column indicate are significantly different (Tukey test, *p* < 0.05).

### 3.9 Water sorption behavior in artificial saliva and disintegration

**Figure 9:**
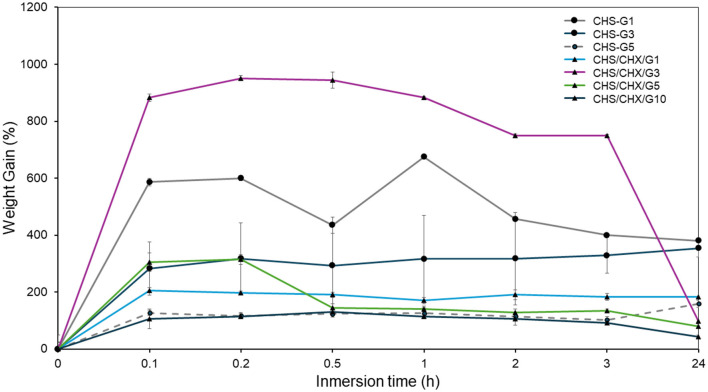
Effect of loading G on water sorption curves and disintegration of CHS/G and CHS/G/CHX membranes in simulated saliva (pH = 7.38) at 35°C, *n* = 3 (mean ± S).

Water sorption is correlated with the hydrophilicity of the membrane (Han et al., 2013). Figure 9 illustrates the percentage of water gains as a function of immersion time for the CHS/G and CHS/G/CHX membranes containing different G loading. The CHS/G 1, 3, 5, and CHS/G/CHX 1, 3, 5, and 10% of G membranes preserved their integrity for 24 h. At the end of the period, the integrity of these membranes began to deteriorate, and they lost water. The CHS/G 10, 15, and 20% membranes with G, and CHS/G/CHX 15 and 20% membranes with G, which were thicker than the other membranes, completely disintegrated within the first 1 min. This G plasticizer is incorporated into CHS chains, promoting the formation of hydrogen bonds between molecules while decreasing the CHS matrix's strong intramolecular attraction (Harussani et al., 2021; Caicedo et al., 2022) as represents tensile strength (Figure 5). The G is a plasticizer that enhances water absorption on matrix CHS. The results of this study made it possible to obtain biodegradable membranes via the casting technique using a formulation with biopolymer CHS with G as a plasticizing agent.

### 3.10 Swelling capacity

In addition, the CHS/G1/CHX and CHS/G3 membranes demonstrated the highest swelling index compared to other membranes, 8.07 ± 0.58 and 7.85 ± 1.07, respectively. Swelling study is a critical parameter that affects the residence time at the application site and the drug release characteristics of membranes. Again, for adhesion, the wettability of the membrane and its swelling ability is of great importance (Arpa et al., 2023). The studies of the swelling behavior of the membrane were conducted in simulated saliva at pH = 7.38 at 37°C. The swelling index of the membranes is show in Table 3. All the membranes grew 3–5 times their initial size. Remarkably, whith the same load of G (1, 3, 5, and 10%), the swelling index of CHS/G and CHS/G/CHX membranes have statistically significant differences (Tukey test, *p* < 0.05). Similar results were obtained by Arpa et al. (2023) with membranes containing chlorhexidine and glycerin as plasticizers and with other biopolymer-based membranes with G (Alasalvar et al., 2023). The hydroxyl groups of G incorporated with the polymer matrix hindered the diffusion of water molecules. Thus, the increase in loading G had an inverse effect on the swelling behavior of the membranes.

### 3.11 The *in vitro* antibacterial activity of membranes

The antibacterial activity was evaluated against *S. mutans*. The agar diffusion method was used to determine the inhibition zone against *S. mutans*. The strain was incubated in Brain Hearth Infusion (BHI) at 37°C for 24h.

#### 3.11.1 Inhibition of *S. mutans* by CHS/G/CHX membranes

The inhibition results against *S. mutans* using CHS/G/CHX membranes revealed that varying concentrations of G did not significantly affect the strain inhibition capacity (Table 4). In all four repetitions conducted, a constant and effective inhibition of *S. mutans* growth was consistently observed. Similar results were obtained by Podolan in 2018, with CHS/G/CHS membranes containing 5% of G and 0.2% of CHX (Podolan et al., 2018). Notably, there was no significant difference between each repetition and the various concentrations of G tested, indicating that G may serve as a foundational element for synthesizing CHS membranes without compromising the inhibitory capacity of the active chitosan ingredient or any antibiotics present in them. Furthermore, it is essential to highlight that G and CHS do not exhibit any inhibitory capacity against *S. mutans* (Table 4), as evidenced by the absence of inhibition zones in the four repetitions of the experiment involving CHS/G-only membranes.

**Table 4:** Inhibition halos formed by CHS/G/CHX membranes with G at different concentrations.

**Membrane**	**R1**	**R2**	**R3**	**R4**	**Average**
CHS/G1/CHX	23	20.86	17.92	21.02	20.70 ± 2.09
CHS/G3/CHX	20.5	18.61	18.39	21.18	19.67 ±1.38
CHS/G5/CHX	16.55	22	17.44	23.05	19.76 ±3.24
CHS/G10/CHX	16.8	20.33	17.3	21.02	18.86 ±2.12
CHS/G1	Negative	Negative	Negative	Negative	Negative

The four repetitions (R) of the experiment are shown. Units are presented in millimeters.

The CFU/ml was determined for each event (Table 5) to ensure consistency in the number of bacteria for each repetition. It is worth noting that the optical density values varied in repetitions 1 and 4; however, this discrepancy did not necessarily indicate a lower bacterial count. It has been reported that relying solely on turbidity as the sole parameter for estimating microbial growth is unreliable because as cells grow, they tend to change shape and accumulate extracellular products in the medium, which can increase optical density without necessarily corresponding to an increase in the actual number of bacteria (Sezonov et al., 2007). Based on the obtained results, it can be affirmed that a consistent number of CFU/ml was maintained throughout the four repetitions, and the inhibition observed in the presence of CHS/G/CHX membranes was effective; suggesting that these membranes could serve as a viable alternative for the treatment of oral pathologies caused by *S. mutans*. This bacterium is considered one of the most important oral pathogenic microorganisms not only because it causes diseases in the oral cavity but also due to its ability to invade different tissues and organs via the bloodstream, promoting systemic diseases (Buonavoglia et al., 2022). Dental caries is one of the most common diseases associated with this microorganism, which if not treated promptly can cause infectious endocarditis in people with heart disease (Nomura et al., 2020). *S. mutans* is also implicated in periodontal diseases and oral squamous cell carcinoma. Its presence is associated with advanced clinical stages and poor disease control, making it a determining factor in the development and progression of oral cancer (Tsai et al., 2022). The use of CHS/G/CHX membranes may help prevent the development of these serious conditions that can compromise human health.

**Table 5:** Log of CFU/ml of *S. mutans* in every repetition.

**Membrane**	**Log of CFU/ml**	**Optical density**
CHS/G1/CHX	8.639 ± 0.11	1.72
CHS/G3/CHX	8.119 ± 0.21	3.57
CHS/G5/CHX	8.243 ± 0.25	3.38
CHS/G10/CHX	8.682 ± 0.12	2.5

The table shows the average of five repetitions.

Plasticizers are substances widely used as polymer additives to improve the physical properties of biopolymer-based membranes (Cobos et al., 2018). G is a naturally occurring 3-carbon alcohol in the human body. This study has demonstrated that loading G to CHS membranes with CHX can be a powerful tool for modifying the properties of membranes with potential clinical applications. It was shown that mechanical properties vary in function of the loading G; for instance, tensile strength decreases with increasing loading G, and elongation increases with increasing loading G. Additionally, constant antibacterial results of CHS/G/CHX membranes against *S. mutans* can be explained due to weak interactions of CHX molecule and matrix copolymeric of CHS through electrostatic and van der Waals forces that allow the permeation of drug delivery. The ion-dipole interactions of the positive charges of CHX with the negative charges of CHS and G explain that CHS/G membranes function as drug carriers. This drug delivery system of CHS/G/CHX at different loading of G can be used for oral mucosal lesions. With respect to the toxicity of G, plasticized chitosan membrane is non-cytotoxicity biocompatibility and biodegradability (Caicedo et al., 2022). The mechanical properties of CHS/CHX membranes are significantly affected by the loading G and can influence the criteria for clinical application.

## 4 Conclusions

The CHS/G and CHS/G/CHX membranes were successfully prepared using CHS as the membrane-forming copolymer in the aqueous acetic solution with different concentrations of G as a plasticizer agent. A transparent homogeneous, clear, and flexible membrane after evaporation was obtained. In conclusion, the loading glycerol at chitosan membranes change the physical properties of membranes. Glycerol at 1 and 3% were found to be the optimal plasticizer for promoting the CHS/G/CHX membrane physical and mechanical properties. The CHS/G/CHX inhibited *in vitro* growth of *S. mutans* CDBB-B-1455. Moreover, toxicity test toward the human cells, as well as antibacterial test against other microorganisms are necessary. It is relevant to mention that CHS/G membranes can function as an effective dispersing agent for different antibiotics or active ingredients that can be used in the treatment of diseases present in the oral cavity. CHS/G/CHX membranes represent an alternative for the treatment of oral diseases caused by *S. mutans*. One of the advantages of using membranes is the reduction of contact with healthy tissues, localizing the treatment to a specific area and thus avoiding side effects in the healthy areas of the oral cavity. This represents an effective alternative to the use of mouthwashes or gels.

## Data availability statement

The original contributions presented in the study are included in the article, further inquiries can be directed to the corresponding authors.

## Author contributions

JH-O: Conceptualization, Investigation, Methodology, Writing – original draft, Funding acquisition, Resources. AJ-D: Conceptualization, Data curation, Formal analysis, Investigation, Methodology, Project administration, Supervision, Writing – original draft, Writing – review & editing, Software, Visualization. LAP-R: Data curation, Formal analysis, Investigation, Writing – review & editing, Writing – original draft. AF-L: Investigation, Methodology, Supervision, Writing – review & editing. DP-G: Data curation, Formal analysis, Software, Writing – review & editing. KP-S: Investigation, Methodology, Project administration, Supervision, Writing – original draft. ER-C: Investigation, Methodology, Writing – review & editing. IJ-D: Investigation, Software, Supervision, Writing – review & editing. MM: Supervision, Writing – review & editing. MG-M: Investigation, Methodology, Project administration, Supervision, Writing – original draft. BC-S: Funding acquisition, Resources, Validation, Visualization, Writing – review & editing. BÁ-C: Supervision, Writing – review & editing, Funding acquisition, Resources, Validation, Visualization. JH-J: Validation, Writing – review & editing, Funding acquisition, Resources, Supervision, Visualization. AR-U: Writing – review & editing, Investigation, Supervision. PG-G: Investigation, Methodology, Writing – review & editing, Validation. MC-S: Methodology, Writing – review & editing.

## References

[B1] Alasalvar H. Yildirim Z. Yildirim M. (2023). Development and characterization of sustainable active pectin films: the role of choline chloride/glycerol-based natural deep eutectic solvent and lavender extracts. Heliyon 9:e21756. 10.1016/j.heliyon.2023.e21756 38034708 PMC10681944

[B2] Arpa M. D. Yagcilar A. P. Biltekin S. N. (2023). Novel benzydamine hydrochloride and chlorhexidine gluconate loaded bioadhesive films for local treatment of buccal infections. J. Drug Deliv. Sci. Technol. 84:104497. 10.1016/j.jddst.2023.104497

[B3] Bartsch S. Kohnert E. Kreutz C. Woelber J. P. Anderson A. Burkhardt A.-S.. (2024). Chlorhexidine digluconate mouthwash alters the oral microbial composition and affects the prevalence of antimicrobial resistance genes. Front. Microbiol. 15:1429692. 10.3389/fmicb.2024.1429692 38983634 PMC11231401

[B4] Buonavoglia A. Trotta A. Camero M. Cordisco M. Dimuccio M. M. Corrente M. (2022). *Streptococcus mutans* associated with endo-periodontal lesions in intact teeth. Appl. Sci. 12:11837. 10.3390/app122211837

[B5] Caicedo C. Díaz-Cruz C. A. Jiménez-Regalado E. J. Aguirre-Loredo R. Y. (2022). Effect of plasticizer content on mechanical and water vapor permeability of maize starch/PVOH/chitosan composite films. Materials 15:41274. 10.3390/ma15041274 35207816 PMC8878178

[B6] Cobos M. González B. Fernández M. J. Fernández M. D. (2018). Study on the effect of graphene and glycerol plasticizer on the properties of chitosan-graphene nanocomposites via in situ green chemical reduction of graphene oxide. Int. J. Biol. Macromol. 114, 599–613. 10.1016/j.ijbiomac.2018.03.129 29588207

[B7] Corral-Lugo A. Morales-García Y. E. Pazos-Rojas L. A. Ramirez-Valverde A. Martínez-Contreras R. D. Muñoz-Rojas J. (2012). Quantification of cultivable bacteria by the ‘Massive Stamping Drop Plate' method. Rev. Colomb. Biotecnol 2, 147–156.

[B8] Dinte E. Muntean D. M. Andrei V. Bo,sca B. A. Dudescu C. M. Barbu-Tudoran L.. (2023). *In vitro* and *in vivo* characterisation of a mucoadhesive buccal film loaded with doxycycline hyclate for topical application in periodontitis. Pharmaceutics 15:580. 10.3390/pharmaceutics15020580 36839899 PMC9963859

[B9] Gao H. Wu N. Wang N. Li J. Sun J. Peng Q. (2022). Chitosan-based therapeutic systems and their potentials in treatment of oral diseases. Int. J. Biol. Macromol. 222, 3178–3194. 10.1016/j.ijbiomac.2022.10.090 36244538

[B10] Guo Y. Qiao D. Zhao S. Liu P. Xie F. Zhang B. (2024). Biofunctional chitosan–biopolymer composites for biomedical applications. Mater. Sci. Eng. R Rep. 159:100775. 10.1016/j.mser.2024.100775

[B11] Han B. Zhang D. Shao Z. Kong L. Lv S. (2013). Preparation and characterization of cellulose acetate/carboxymethyl cellulose acetate blend ultrafiltration membranes. Desalination 311, 80–89. 10.1016/j.desal.2012.11.002

[B12] Harussani M. M. Sapuan S. M. Firdaus A. H. M. El-Badry Y. A. Hussein E. E. El-Bahy Z. M. (2021). Determination of the tensile properties and biodegradability of cornstarch-based biopolymers plasticized with sorbitol and glycerol. Polymers 13:3709. 10.3390/polym13213709 34771264 PMC8587433

[B13] Hemmingsen L. M. Giordani B. Pettersen A. K. Vitali B. Basnet P. Škalko-Basnet N. (2021). Liposomes-in-chitosan hydrogel boosts potential of chlorhexidine in biofilm eradication in vitro. Carbohydr. Polym. 262:117939. 10.1016/j.carbpol.2021.117939 33838816

[B14] Hemmingsen L. M. Panchai P. Julin K. Basnet P. Nystad M. Johannessen M.. (2022). Chitosan-based delivery system enhances antimicrobial activity of chlorhexidine. Front. Microbiol. 13:1023083. 10.3389/fmicb.2022.1023083 36246245 PMC9557914

[B15] Hubbard A. T. M. Coates A. R. M. Harvey R. D. (2017). Comparing the action of HT61 and chlorhexidine on natural and model Staphylococcus aureus membranes. J. Antibiot. 70, 1020–1025. 10.1038/ja.2017.90 28765589

[B16] Korelc K. Larsen B. S. Gašperlin M. Tho I. (2023). Water-soluble chitosan eases development of mucoadhesive buccal films and wafers for children. Int. J. Pharm. 631:122544. 10.1016/j.ijpharm.2022.122544 36572261

[B17] Kusmono Abdurrahim I. (2019). Water sorption, antimicrobial activity, and thermal and mechanical properties of chitosan/clay/glycerol nanocomposite films. Heliyon 5:e02342. 10.1016/j.heliyon.2019.e02342 31485529 PMC6717162

[B18] Ładniak A. Jurak M. Wiacek A. E. (2021). Physicochemical characteristics of chitosan-TiO2 biomaterial. 2. Wettability and biocompatibility. Colloids Surf. A Physicochem. Eng. Aspects 630:127546. 10.1016/j.colsurfa.2021.127546

[B19] Mohammadi Z. Eini M. Rastegari A. Tehrani M. R. (2021). Chitosan as a machine for biomolecule delivery: a review. Carbohydr. Polym. 256:117414. 10.1016/j.carbpol.2020.117414 33483009

[B20] Nilsson M. Jakobsen T. H. Givskov M. Twetman S. Tolker-Nielsen T. (2019). Oxidative stress response plays a role in antibiotic tolerance of *Streptococcus mutans* biofilms. Microbiology 165, 334–342. 10.1099/mic.0.000773 30663959

[B21] Nomura R. Matayoshi S. Otsugu M. Kitamura T. Teramoto N. Nakano K. (2020). contribution of severe dental caries induced by *Streptococcus MUTANS* to the pathogenicity of infective endocarditis. Infect. Immun. (2020) 88:e00897–19. 10.1128/IAI.00897-19 32312765 PMC7309618

[B22] Podolan K. A. Lourenço N. N. da Aparecida C. S. Marchini O. T. de Cardoso O. R. Aparecida A. M. M. M.. (2018). *In vitro* antimicrobial effect of bioadhesive oral membrane with chlorhexidine gel. Braz. Dent. J. 29, 354–358. 10.1590/0103-6440201801743 30462761

[B23] Polizzi E. Tetè G. Bova F. Pantaleo G. Gastaldi G. Capparè P.. (2020). Antibacterial properties and side effects of chlorhexidinebased mouthwashes. A prospective, randomized clinical study. J. Osseointegr. 12, 1–7. 10.23805/JO.2019.12.01.20

[B24] Ren J. Dang K. M. Pollet E. Avérous L. (2018). Preparation and characterization of thermoplastic potato starch/halloysite nano-biocomposites: effect of plasticizer nature and nanoclay content. Polymers 10:808. 10.3390/polym10080808 30960733 PMC6403770

[B25] Sezonov G. Joseleau-Petit D. D'Ari R. (2007). *Escherichia coli* physiology in Luria-Bertani broth. J. Bacteriol. 189, 8746–8749. 10.1128/JB.01368-07 17905994 PMC2168924

[B26] Shojaee Kang Sofla M. Mortazavi S. Seyfi J. (2020). Preparation and characterization of polyvinyl alcohol/chitosan blends plasticized and compatibilized by glycerol/polyethylene glycol. Carbohydr. Polym. 232:115784. 10.1016/j.carbpol.2019.115784 31952592

[B27] Stachowiak N. Kowalonek J. Kozlowska J. (2020). Effect of plasticizer and surfactant on the properties of poly(vinyl alcohol)/chitosan films. Int. J. Biol. Macromol. 164, 2100–2107. 10.1016/j.ijbiomac.2020.08.001 32758608

[B28] Sun Y. Liu Z. Zhang L. Wang X. Li L. (2020). Effects of plasticizer type and concentration on rheological, physico-mechanical and structural properties of chitosan/zein film. Int. J. Biol. Macromol. 143, 334–340. 10.1016/j.ijbiomac.2019.12.035 31812748

[B29] Tarique J. Sapuan S. M. Khalina A. (2021). Effect of glycerol plasticizer loading on the physical, mechanical, thermal, and barrier properties of arrowroot (*Maranta arundinacea*) starch biopolymers. Sci. Rep. 11:13900. 10.1038/s41598-021-93094-y 34230523 PMC8260728

[B30] Terukina T. Tanaka J. Takayama Y. Osanai K. Kino S. Kanazawa T.. (2023). Sangelose-based gels and films: effects of glycerol and α-cyclodextrin and their pharmaceutical application. Drug Dev. Ind. Pharm. 49, 75–83. 10.1080/03639045.2023.2182127 36803493

[B31] Tsai M.-S. Chen Y.-Y. Chen W.-C. Chen M.-F. (2022). *Streptococcus mutans* promotes tumor progression in oral squamous cell carcinoma. J. Cancer 13, 3358–3367. 10.7150/jca.73310 36186905 PMC9516012

[B32] Tsuda H. Yamashita Y. Shibata Y. Nakano Y. Koga T. (2002). Genes involved in bacitracin resistance in *Streptococcus mutans*. Antimicrob. Agents Chemother. 46, 3756–3764. 10.1128/AAC.46.12.3756-3764.2002 12435673 PMC132740

[B33] Wang B. Xu X. Fang Y. Yan S. Cui B. Abd El-Aty A. M. (2022). Effect of different ratios of glycerol and erythritol on properties of corn starch-based films. Front. Nutr. 9:882682. 10.3389/fnut.2022.882682 35548578 PMC9083458

[B34] Xu X. Wang B. Gao W. Sui J. Wang J. Cui B. (2023). Effect of different proportions of glycerol and D-mannitol as plasticizer on the properties of extruded corn starch. Front. Nutr. 10:1335812. 10.3389/fnut.2023.1335812 38299182 PMC10829104

[B35] Yadav N. Mudgal D. Anand R. Jindal S. Mishra V. (2022). Recent development in nanoencapsulation and delivery of natural bioactives through chitosan scaffolds for various biological applications. Int. J. Biol. Macromol. 220, 537–572. 10.1016/j.ijbiomac.2022.08.098 35987359

